# Exome-Based Identification of Candidate Genes in Sporadic Adenomyosis Cases

**DOI:** 10.3390/diagnostics15233069

**Published:** 2025-12-02

**Authors:** Feyza Nur Tuncer, Nimet Eser Ma, Sevcan Aydin, Nura Fitnat Topbas Selcuki, Ipek Yildiz Ozaydin, Engin Oral

**Affiliations:** 1 Department of Genetics, Aziz Sancar Institute of Experimental Medicine, Istanbul University, 34093 Istanbul, Türkiye; 2 Graduate School of Health Sciences, Istanbul University, 34126 Istanbul, Türkiye; nimeteser1@gmail.com (N.E.M.); aydin.sevcan.20@ogr.iu.edu.tr (S.A.); 3 Department of Obstetrics and Gynecology, Istanbul Sisli Hamidiye Etfal Training and Research Hospital, University of Health Sciences Türkiye, 34453 Istanbul, Türkiye; fitnat.topbas@gmail.com; 4 Department of Pathology, Istanbul Kanuni Sultan Suleyman Training and Research Hospital, University of Health Sciences Türkiye, 34303 Istanbul, Türkiye; dripekyildizozaydin@gmail.com; 5 Department of Clinical Sciences Obstetrics and Gynaecology Unit, Norrland University Hospital, Umeå University, 901 85 Umeå, Sweden; drenginoral@gmail.com

**Keywords:** adenomyosis, whole exome sequencing, *EFHB*, *MEIS1*

## Abstract

**Background**: Adenomyosis is a benign uterine disorder defined by the invagination of ectopic endometrial-like tissue into the myometrium, causing heavy menstrual bleeding and pain. While its pathogenesis remains unclear, shared-symptomology with endometriosis suggests a common mechanism. Adenomyosis is often diagnosed after age 40 due to its complex presentation and the need for histopathological confirmation, underscoring the need for non-invasive markers. **Methods**: Ten unrelated women with histopathological diagnosis of adenomyosis were recruited. All recruits completed the WERF-EPHect questionnaire and were additionally questioned about any comorbidities. Genomic DNA isolated from peripheral blood was subjected to whole exome sequencing (WES) on Illumina NovaSeq 6000 and was analyzed using the Pairend NGS Cloud platform. Variants were filtered for MAF < 1% and were prioritized based on functional relevance and impact determined by *in silico* prediction tools. Variant selection adhered to stringent quality metrics to identify candidate variants associated with adenomyosis. **Results**: WES analysis did not reveal any variant common to the cohort. A total of eight pathogenic and two likely pathogenic novel variants were identified. Moreover, novel variants of p.(Val331Ile) in *EFHB* and p.(Phe14Val) in *MEIS1* were the most frequently shared genetic variants in the cohort. **Conclusions**: Our findings suggest novel candidate genes for adenomyosis that warrant validation and functional investigation in larger, independent cohorts.

## 1. Introduction

Adenomyosis is a benign gynecological condition characterized by the ectopic presence of endometrial glands and stroma within the myometrium, resulting in reactive hyperplastic and hypertrophic changes [1]. While approximately one-third of affected individuals remain asymptomatic, the predominant clinical manifestations include menorrhagia, dysmenorrhea, chronic pelvic pain, dyspareunia, and infertility [2]. The pathogenesis of adenomyosis remains elusive, although several hypotheses—including endometrial invasion into the myometrium, microtrauma at the myometrial–endometrial interface, and de novo metaplasia—have been proposed to explain this enigmatic condition [3]. Owing to the complex nature of the disease, the heterogeneity of clinical presentation, and the requirement of histopathological confirmation for a definitive diagnosis, most cases are diagnosed after the age of 40 years. Furthermore, since nearly one-third of affected individuals may remain asymptomatic, this substantially contributes to heterogeneity among patients, further complicating both early detection and accurate diagnosis. Together, these factors underscore the challenges in defining the true clinical and molecular spectrum of adenomyosis, with an average diagnostic delay of 11 years [4,5].

Though distinct conditions, patients frequently exhibit both adenomyosis and endometriosis. Endometriosis, as an estrogen-induced benign gynecological condition, is described by the presence of endometrial-like tissues outside the uterus [6]. Due to the presence of shared symptoms, including chronic pelvic pain, dyspareunia and infertility, a common pathogenesis between these conditions has been considered [5]. When comparing the extent of genetic analyses performed in patients diagnosed with either condition, endometriosis has been studied more extensively; a genome-wide association study (GWAS) meta-analysis revealed 42 genetic loci explaining approximately 5% of disease variance [7]. Despite its relatively high prevalence, affecting approximately 10% of women of reproductive age, endometriosis patients may still receive a definitive diagnosis only after delays of up to 12 years, underscoring the complex and heterogeneous nature of the condition [8].

This study sought to address the current gap in limited genetic research on adenomyosis by utilizing whole-exome sequencing (WES) to identify genes potentially involved in its pathogenesis, while also drawing insights from the more extensively studied genetic background of endometriosis.

## 2. Methods

### 2.1. Patients and Clinical Assessment

Ten unrelated women who underwent hysterectomy for abnormal uterine bleeding and were diagnosed with adenomyosis upon histopathological examination were retrospectively recruited by reviewing their medical records. All recruits had a family history of adenomyosis or its related symptoms and were invited for an interview to complete any missing clinical data. Sample collection was commenced after obtaining written informed consent in accordance with the Istanbul Medical Faculty Clinical Research Ethics Committee (Protocol no: 2023/1504). All cases completed the Turkish-adapted version of the World Endometriosis Research Foundation–Endometriosis Phenome Harmonisation Project (WERF-EPHect) questionnaire [9,10], which assessed abnormal uterine bleeding (menorrhagia, metrorrhagia, oligomenorrhea, polymenorrhea, and menstrual duration) and pain severity (dysmenorrhea, chronic pelvic pain, and dyspareunia) on a scale of ten, and were retrospectively screened and included. The questionnaire also contained additional questions assessing the presence of comorbidities, including cancer, metabolic disorders, endocrinological conditions, cardiovascular diseases, and autoimmune disorders.

‘World Endometriosis Research Foundation Endometriosis Phenome and Biobanking Harmonization Project: III. Fluid biospecimen collection, processing, and storage in endometriosis’ standard operating procedures were followed [11]. Genomic DNA was extracted from peripheral blood using the PureLink Genomic DNA Mini Kit (Thermo Fisher Scientific, Inc., Waltham, MA, USA). The quantity and purity of genomic DNA were measured with a NanoDrop™ 2000 Spectrophotometer (Thermo Fisher Scientific, Waltham, MA, USA) and an Invitrogen™ Qubit™ 3 Fluorometer (Thermo Fisher Scientific, Inc., Waltham, MA, USA) using Invitrogen™ Qubit™ dsDNA Quantification Assay Kits (Thermo Fisher Scientific, Inc., Waltham, MA, USA).

### 2.2. Whole Exome Sequencing (WES)

The Illumina Nextera DNA Exome (Illumina, San Diego, CA, USA) was used for library preparation on all subjects. The kit targeted 45 Mb of protein-coding regions, covering > 99% of RefSeq, CCDS, and GENCODE databases, and was subsequently sequenced on Illumina NovaSeq 6000 system to achieve a minimum of 50X read depth for the targeted bases. Variant annotations and subsequent filtering were achieved utilizing an artificial intelligence based data analysis platform NGS Cloud (www.ngscloud.com; Pairend Biotechnology LLC, İstanbul, Türkiye), as described previously [12]. 

Variant prioritizations were performed for minor allele frequencies (MAF) <1% in all normal populations, with intronic and synonymous variants filtered out. A stepwise variant selection strategy was employed to systematically list candidate genetic variants based on their associations with endometriosis/adenomyosis disorders in existing literature [13], the functional relevance of the genes, and/or the predicted pathogenicity of the variants. Selected variants were subjected to stringent quality metrics prior to being listed as candidates. These parameters included a minimum read depth of 30X, a variant allele fraction > 0.25, a base quality score > 30, and absence of significant strand bias. Variants fulfilling these criteria were classified as high-confidence calls and listed as candidate variants for adenomyosis.

Initially, the cohort was screened for any shared genetic variants. Following this initial step, cases were evaluated on an individual basis to generate a list of candidate genes. The IGV_2.9.4 program was used to visualize sequence reads. MAFs were obtained from GnomAD and the NGS Cloud *in house* database, comprising approximately 30,000 exome sequences of individuals from Türkiye with varying disorders, given the lack of a population-specific genetic database for Türkiye. Variants were interpreted in accordance with the American College of Medical Genetics Guidelines (ACMG) and the ClinVar Database.

## 3. Results

### 3.1. Clinical Findings

The cohort consisted of patients over 45 years of age. All were diagnosed with adenomyosis only and had no comorbidities. Pregnancy histories indicated that all cases had experienced at least two gravidae. Histories of curettage and/or abortus were present in all patients, and none reported ectopic pregnancy. The most remarkable case was Case 255, with 12 gravidae, 6 abortuses, and 2 curettages. Overall, all cases had been exposed to intrauterine surgical interventions (Table 1).

The results of the WERF-EPHect questionnaire are represented in Table 2 and Table 3. Menstrual bleeding information for Cases 262 and 263 is unknown, while Case 264 provided information on menorrhagia and metrorrhagia (Table 2). Table 3 shows information on pain and other main symptoms, including abnormal menstrual bleeding, chronic pelvic pain, dysmenorrhea, and dyspareunia (pain during or after intercourse), as well as the age of onset, severity, and treatment methods for these symptoms.

Among the patients with dysmenorrhea, all cases described their pain as severe. Only Cases 250 and 260 did not report dysmenorrhea. All patients, except for Cases 250 and 264, presented with chronic pelvic pain, and only half of the cohort described pain during or after sexual intercourse (Table 3).

### 3.2. Genetic Findings

WES analysis did not reveal a shared genetic variant across all cases; however, individual analyses identified a total of eight pathogenic and two likely pathogenic variants (Table 4 and Table 5). Moreover, four cases (250, 260, 263, 264) shared the *EFHB* NM_144715:c.990_991delinsGA, p.(Val331Ile) [rs386659061] variant, while the *MEIS1* NM_002398:c.39T>G, p.(Phe14Val) variant was shared among three cases (250, 254, 260).

## 4. Discussion

Adenomyosis is a benign uterine disorder, but it can considerably impair women’s health and quality of life, depending on the severity of the associated symptoms, which vary among affected individuals. Accordingly, our evaluation of genetic variants primarily focused on bleeding and pain, as these are the main symptoms that drive diagnosis and medical consultation in adenomyosis. Herein, we prioritized discussing the plausible involvement of the shared genes in adenomyosis pathogenesis, followed by an evaluation of pathogenic variants determined in cases with the severe symptoms mentioned.

Due to the multifactorial nature of adenomyosis, our findings revealed many candidate genes, as expected. Among these, p.(Val331Ile) in *EFHB* and p.(Phe14Val) in *MEIS1* were the most frequently shared genetic variants in the cohort, supporting their potential involvement in disease pathogenesis. (Table 4). Of the two patient groups carrying these variants, all but Cases 250 and 260 reported severe pain related to adenomyosis. *EFHB* has been associated with calcium regulation and encodes a protein that acts as a regulator in store-operated Ca^2+^ entry (SECO). Previous studies have shown that changes in calcium balance in SECO-derived cells cause cellular migration in disorders such as cancer [14]. The gene also has been found to have regulatory roles in proliferation and apoptosis [15]. All these features suggest that it may affect invagination of the endometrium into the myometrium. Increased invagination may contribute to heightened vascularization and hormonal changes due to increased tissue volume, suggesting that the *EFHB* gene may play a role in abnormal uterine bleeding (AUB) [16]. Furthermore, considering that calcium balance directly affects the regulation of myometrial contractions [17], it might contribute to abnormal contractions in the myometrium and abnormal menstrual bleeding during the menstrual cycle. On the other hand, *MEIS1* acts as a cofactor for HOXA10 in the human endometrium, a homeobox-containing transcription factor that plays a role in both uterine development during the embryonic process and endometrial development during the menstrual cycle in adulthood. It is one of the important genes that was previously implicated in adenomyosis [18]. Studies have observed that *HOXA10* gene expression is significantly downregulated in the endometrium of women with both adenomyosis and endometriosis [19,20]. Consistently, *MEIS1* expression was decreased in endometrial stromal cells from endometriosis patients as well as in a mouse model of the disease. Moreover, *MEIS1* suppression enhanced the proliferative capacity of endometrial stromal cells. Taken together, these results suggest that loss of *MEIS1* expression may disrupt the balance between apoptosis and proliferation in endometrial stromal cells, thereby favoring disease development [21]. Collectively, it can be deduced that *MEIS1* may affect implantation difficulties and endometrial proliferations, aligning with the invagination hypothesis for adenomyosis.

Given that adenomyosis is a complex disorder influenced by multiple genetic and environmental factors, we selected a sporadic cohort of 10 affected women, whose family members were either diagnosed with adenomyosis or exhibited related symptoms. This approach aimed to correlate the pathogenic variants identified through individual WES analysis of each patient in the cohort. Due to the limited size of our cohort, the novel pathogenic gene variants presented in Table 5 should be regarded as preliminary candidates; their association with adenomyosis is currently inferred solely from their known biological functions, rendering them hypothetical at this stage. Therefore, the following paragraphs are intended to elaborate the rationale for proposing their potential involvement in adenomyosis pathogenesis, thereby paving the way for future research.

In the evaluation of bleeding and pain symptoms of adenomyosis in our cohort, Case 255 exhibited the heaviest bleeding with prolonged menstruation and pain occurring chronically as well as before and after sexual intercourse (Table 3). Familial transition of AUB was present in the case’s aunt, older sister, and daughter, who refused to contribute their DNA samples for the study. Among the five novel genetic variants detected in Case 255, *SULT2B1* [NM_17797:c.553C>T, p.(Gln185Ter)] is the only pathogenic one reported. As an extensively studied gene involved in disparate conditions, its downregulation has been implicated in promoting the proliferation of ectopic endometrial cells in ovarian endometriosis tissues [22]. It synthesizes two enzymes that have been linked to the homeostasis of sex hormones, and SULT2B1b was found at high-levels in the brain; this might be one of the genes involved in pain perception among adenomyosis patients [23]. Moreover, overexpression of *SULT2B1* has been correlated with poor prognosis in endometrial, cervical, and ovarian cancers [23,24,25,26,27], thereby representing a potential link that merits future evaluation regarding the coexistence of adenomyosis with ovarian cancer, as implicated in the literature [28]. The remaining four novel genetic variants were classified as variants of uncertain significance (VUS); nevertheless, their potential contribution to disease pathogenesis and symptom severity cannot be excluded.

When the genetic results of the remaining cases in the cohort were evaluated, five pathogenic novel variants—*CYP27A1* [NM_00078:c.808C>T, p.(Arg270Ter)], *ERCC2* [NM_000400:c.1846C>T, p.(Arg616Trp)], *HGFAC* [NM_001528:c.480_495del, p.(Leu161ProfsTer81)], *CTSK* [NM_000396:c.721C>T, p.(Arg241Ter)], and *ADAM15* [NM_207197:c.2278C>T, p.(Gln760Ter)]—were determined (Table 5). Among these genes, polymorphisms in *CYP27A1* has been suggested to affect vitamin D metabolism [29]. In line with this, a recent study involving a total of 336 women suggested the association between low vitamin D levels and the onset of adenomyosis [30], supporting the involvement of *CYP27A1* in this complex condition. The impact of *ERCC2* polymorphisms, a helicase involved in nucleotide excision repair, has been extensively investigated in various cancers, including gynecological tumors [31]. Certain variants in this gene have also been linked to the development of endometriosis [32], suggesting a potential role for *ERCC2* in adenomyosis within the context of shared pathogenesis. The termination variant detected in *Hepatocyte Growth Factor Activator* (*HGFAC*) highlights it as a plausible candidate gene for adenomyosis, given its role in activating HGF, which in turn promotes epithelial–mesenchymal transition (EMT) and drives glandular invagination into the myometrium [33]. On the other hand, as a candidate diagnostic biomarker, *CTSK* has been depicted as one of the players in inflammation-related pathways in recent studies involving women with endometriosis [34,35]. The novel *ADAM15* variant, resulting in premature protein termination, merits functional evaluation to assess its reported impact on intrauterine adhesions [36], potentially clarifying its involvement in menstrual bleeding and adenomyosis pathogenesis. Among the cases in the cohort, notable accumulation of pathogenic variants was detected in Case 264, which might be associated with an early age of onset for dysmenorrhea. The remaining genes detected in this cohort are likely to exert an undeniable cumulative effect on the pathogenesis of a complex disease such as adenomyosis, underscoring the need for validation in independent cohorts as well as mechanistic insights from functional studies.

Our study is limited by the small sample size; however, it represents a pioneering effort in identifying candidate genes for adenomyosis using WES in a cohort of histopathologically confirmed cases. The lack of a shared genetic variant among patients diagnosed solely with adenomyosis and without comorbidities highlights the complexity of this condition. Nevertheless, it should be noted that despite efforts to detect comorbities, the risk for metabolic disorders cannot be accurately assessed, as recruitment was conducted retrospectively. However, this limitation does not directly affect the genetic analyses of our cohort since gene expression levels were not examined. Thereby, while our findings are promising, they should be interpreted with caution and require validation in larger, independent cohorts. Furthermore, functional studies are essential to clarify the underlying mechanisms, ultimately contributing to a better understanding of this complex condition.

## Figures and Tables

**Table 1:** Demographic and clinical information of the study cohort.

Case ID	Age	BMI	Obstetric History	Cesarean Section	Infertility/Pregnancy Treatment	Non-Hormonal IUD ** Use	Hormone Use	Surgical History
			(G/P/A/C/E) *					
250	63	28	6/4/0/2/0	No	No	Yes	No	Curettage
251	51	25	3/2/0/1/0	Yes	No	Yes	No	Curettage, Tubal ligation
253	53	26	5/3/2/0/0	No	No	Yes	No	Oophorectomy
254	49	26	3/2/0/1/0	No	No	No	No	Curettage
255	49	28	12/4/6/2/0	No	No	No	No	Curettage, Myomectomy, Oophorectomy, Tubal ligation
260	59	27	5/5/0/1/0	No	No	Yes	No	Curettage
261	47	25	2/2/0/1/0	No	No	Yes	No	Curettage
262	68	28	4/3/0/1/0	No	No	N/A	N/A	Curettage
263	53	28	4/2/1/1/0	No	No	N/A	No	Curettage, Bilateral oophorectomy
264	46	27	4/2/2/0/0	No	No	Yes	No	Myomectomy

* G, Gravida (number of pregnancies); P, Parity (number of births); A, Abortion (number of miscarriages); C, Curettage; E, Ectopic pregnancy; ** IUD, Intrauterine Device; N/A, Not Available.

**Table 2:** Menstrual bleeding information of the cohort.

Case	Menorrhagia *	Metrorrhagia	Oligomenorrhea	Polymenorrhea	Menstrual Duration (day)
250	Yes (4/10)	No	No	No	5
251	Yes (10/10)	No	No	No	10
253	Yes (10/10)	Yes	No	Yes	10
254	Yes (10/10)	Yes	Yes	Yes	10
255	Yes (10/10)	Yes	No	Yes	≥10
260	Yes (10/10)	Yes	No	No	10
261	Yes (10/10)	No	No	No	10
262	NA	NA	NA	NA	NA
263	NA	NA	NA	NA	NA
264	Yes (10/10)	Yes	NA	NA	NA

* Assessed on a scale from 1 to 10 (10 representing the heaviest). NA (not available).

**Table 3:** Pain information of the cohort.

Case	Dysmenorrhea/ AO (Years)	Pelvic Pain During Menstruation *	Chronic Pelvic Pain */ AO (Years)	Dyspareunia	Pain During or After Sexual Intercourse	Dyspareunia AO (Years)	Severity of Dyspareunia
250	No	1/10	No	No	No	No	No
251	Yes 15	10/10	10/10 15	Yes	During	45	10/10
253	Yes 44	10/10	3/10 NA	Yes	During & After	NA	5/10
254	Yes 18	10/10	10/10 44	Yes	After	NA	10/10
255	Yes 40	10/10	8/10 46	Yes	During & After	NA	8/10
260	No	1/10	4/10 NA	Yes	During	NA	3/10
261	Yes NA	10/10	7/10 NA	No	No	No	No
262	Yes 17	10/10	5/10 40	No	No	No	No
263	Yes 21	10/10	6/10 50	No	No	No	No
264	Yes 13	10/10	NA	NA	NA	NA	NA

* Assessed on a scale from 1 to 10 (10 representing the severest); NA, Not available; AO, Age of Onset.

**Table 4:** Shared genes of the cohort.

Gene	Case	Variant	Consequence	SNP ID	Frequency (GenomAD/ *In House*)	ACMG
*EFHB* NM_144715	250	c.990_991delinsGA	p.(Val331Ile)	rs386659061	0.0000%/0.0000%	ACMG_VUS
	253	c.2198G>A	p.(Arg733Gln)	rs149572350	0.2384%/0.2212%	ACMG_VUS
	260	c.990_991delinsGA	p.(Val331Ile)	rs386659061	0.0000%/0.0000%	ACMG_VUS
	263	c.990_991delinsGA	p.(Val331Ile)	rs386659061	0.0000%/0.0000%	ACMG_VUS
	264	c.990_991delinsGA	p.(Val331Ile)	rs386659061	0.0000%/0.0000%	ACMG_VUS
*MEIS1* NM_002398	250	c.39T>G	p.(Phe14Val)	-	0.0000%/0.0000%	ACMG_VUS
	254	c.39T>G	p.(Phe14Val)	-	0.0000%/0.0000%	ACMG_VUS
	260	c.39T>G	p.(Phe14Val)	-	0.0000%/0.0000%	ACMG_VUS
	263	c.60del	p.(Ser21ProfsTer19)	-	0.0000%/0.0000%	ACMG_P
		c.54_55del	p.(Ile19ProfsTer50)	-	0.0000%/0.0000%	ACMG_P

All identified genetic changes were heterozygous. VUS, Variant of Uncertain Significance; P, Pathogenic.

**Table 5:** Preliminary WES-identified candidate variants of individual cases hypothesized to contribute to adenomyosis pathogenesis.

Case	Gene	Variant	Consequence	SNP ID	Frequency (GenomAD/ *In House*)	ACMG/ ClinVar
250	*ANTXR2* NM_001145794	c.1068_1069delinsAC	p.(Ala357Pro)	rs386676514	0.0000%/0.0000%	ACMG_VUS
	*CYP27A1* NM_000784	c.808C>T	p.(Arg270Ter)	rs72551318	0.0123%/0.0000%	ClinVar_P ACMG_P
	*CTNNA1* NM_001903	c.376C>T	p.(Arg126Trp)	-	0.0000%/0.0000%	ClinVar_VUS ACMG_VUS
251	*KIR3DL1* NM_0132892	c.243C>A	p.(Asn81Lys)	rs190589393	0.0037%/0.0000%	ACMG_VUS
	*NDUFA13* NM_015965	c.220C>T	p.(Pro74Ser)	rs377613939	0.0425%/0.0000%	ACMG_VUS
	*MMP3* NM_002422	c.654T>G	p.(His218Gln)	rs557512280	0.0163%/0.0000%	ACMG_VUS
	*CYP27A1* NM_000784	c.215T>A	p.(Leu72Gln)	rs138189735	0.0326%/0.0000%	ClinVar_VUS ACMG_VUS
253	*ZC3H13* NM_015070	c.1049G>A	p.(Arg350His)	rs11537603	0.3448%/0.0000%	ClinVar_VUS
	*IL18R1* NM_003855	c.1112-5C>T	-	-	0.0000%/0.0000%	ACMG_VUS
	*ITGA2B* NM_000419	c.457G>A	p.(Ala153Thr)	rs199641871	0.1179%/0.0000%	ClinVar_VUS ACMG_VUS
	*PPP3CB* NM_021132	c.674A>G	p.(Asp225Gly)	rs1472878143	0.0000%/0.0000%	ACMG_LP
254	*CTBP1* NM_001328	c.1270G>A	p.(Gly424Ser)	-	0.0000%/0.2212%	ACMG_VUS
	*FGA* NM_000508	c.923G>A	p.(Arg308Gln)	rs760992799	0.0054%/0.0000%	ClinVar_VUS ACMG_VUS
255	*VPS13B* NM_017890	c.1484C>A	p.(Thr495Lys)	-	0.0000%/0.0000%	ACMG_VUS
	*VEZT* NM_017599	c.2338dup	p.(Ter780LeufsTer9)	rs780796608	0.0010%/0.0000%	ACMG_VUS
	*SULT2B1* NM_177973	c.553C>T	p.(Gln185Ter)	-	0.0000%/0.0000%	ACMG_P
	*ANTXR2* NM_001145794	c.746G>A	p.(Arg249Gln)	rs764149126	0.0179%/0.0000%	ClinVar_VUS ACMG_VUS
	*HNRNPM* NM_005968	c.346G>A	p.(Trp116Ter)	rs1313233334	0.0018%/0.0000%	ACMG_VUS
260	*ERCC2* NM_000400	c.1846C>T	p.(Arg616Trp)	rs121913024	0.0115%/0.0000%	ClinVar_P
261	*TSC2* NM_000548	c.2584G>A	p.(Ala862Thr)	rs759837836	0.0230%/0.0000%	ClinVar_CIOP ACMG_VUS
	*NOTCH1* NM_017617	c.3905G>A	p.(Arg1302His)	rs762091081	0.0145%/0.0000%	ClinVar_VUS ACMG_VUS
	*PLXND1* NM_015103	c.4310C>T	p.(Ala1437Val)	rs762492313	0.0130%/0.0000%	ClinVar_VUS ACMG_VUS
	*HPSE2* NM_021828	c.211G>A	p.(Val71Ile)	rs745645944	0.0919%/0.0000%	ClinVar_VUS ACMG_VUS
262	*TSC2* NM_000548	c.251C>T	p.(Ala84Val)	rs35660529	0.3448%/0.0000%	ClinVar_CIOP ACMG_VUS
	*TH* NM_199292	c.1411G>T	p.(Ala471Ser)	rs374465917	0.0163%/0.0000%	ClinVar_VUS ACMG_VUS
	*HGFAC* NM_001528	c.480_495del	p.(Leu161ProfsTer81)	rs562047823	0.6986%/0.4425%	ACMG_P
263	*CYP4F3* NM_000896	c.835C>A	p.(Pro279Thr)	rs774001285	0.0046%/0.0000%	ACMG_VUS
	*MYO7A* NM_000260	c.562C>G	p.(Gln188Glu)	rs572959359	0.1284%/0.0000%	ACMG_VUS ClinVar_CIOP
264	*VEZT* NM_017599	c.2182C>T	p.(Arg728Trp)	rs200245166	0.0166%/0.0000%	ACMG_VUS
	*CTSK* NM_000396	c.721C>T	p.(Arg241Ter)	rs74315303	0.0326%/0.0000%	ClinVar_P ACMG_P
	*ADAM15* NM_207197	c.2278C>T	p.(Gln760Ter)	-	0.0000%/0.0000%	ACMG_P
	*GATAD2B* NM_020699	c.1766T>A	p.(Ile589Asn)	-	0.0000%/0.0000%	ACMG_LP

Significance; P, Pathogenic; LP, Likely Pathogenic; VUS, Variant of Uncertain Significance.

## Data Availability

The datasets generated and/or analyzed during the current study are not publicly available due to the Personal Data Protection Law (KVKK) in Türkiye, but are available from the corresponding author upon a reasonable request.
